# BCL-6 suppresses miR-142-3p/5p expression in SLE CD4^+^ T cells by modulating histone methylation and acetylation of the miR-142 promoter

**DOI:** 10.1038/s41423-019-0268-3

**Published:** 2019-08-20

**Authors:** Shu Ding, Qing Zhang, Shuangyan Luo, Lihua Gao, Jinhua Huang, Jianyun Lu, Jing Chen, Qinghai Zeng, Aiyuan Guo, Jinrong Zeng, Qianjin Lu

**Affiliations:** 1 Department of Dermatology, The Third Xiangya Hospital of Central South University, #138 Tong Zipo Road, 410013 Changsha, Hunan China; 2 Department of Dermatology, The Second Xiangya Hospital of Central South University, #139 Renmin Middle Road, 410011 Changsha, Hunan China

**Keywords:** SLE, BCL-6, EZH2, HDAC5, miR-142-3p/5p, Autoimmunity, Epigenetics in immune cells

## Abstract

The reduced expression of miR-142-3p/5p in CD4^+^ T cells of SLE patients caused T cell hyperactivity and B cell hyperstimulation. This study aimed to investigate the mechanisms of regulating miR-142-3p/5p expression in SLE CD4^+^ T cells. The BCL-6 expression was significantly increased in SLE CD4^+^ T cells compared with normal controls, and the BCL-6 expression was inversely correlated with miR-142-3p/5p expression. BCL-6 suppresses the expression of miR-142-3p/5p by increasing H3K27me3 level and reducing H3K9/K14ac levels in SLE CD4^+^ T cells. BCL-6 regulates histone modifications in miR-142 promoter by recruiting EZH2 and HDAC5. Furthermore, we observed significantly decreased CD40L, ICOS, and IL-21 expression levels in SLE CD4^+^ T cells with BCL-6 interference, and obviously reduced autoantibody IgG production in autologous B cells co-cultured with BCL-6 inhibited SLE CD4^+^ T cells. Our study found that increased BCL-6 up-regulates H3K27me3 and down-regulates H3K9/14ac at miR-142 promoter in SLE CD4^+^ T cells. These factors induce a declination in miR-142-3p/5p expression, consequently resulting in CD4^+^ T cell hyperactivity.

## Introduction

Systemic lupus erythematosus (SLE) is a complicated autoimmune disease characterized by the loss of tolerance towards nuclear components. This loss of tolerance subsequently leads to uncontrolled T cell overactivation and the overproduction of autoantibodies against multiple self-antigens that trigger inflammation and damage to tissues including the skin, joints, vessels, kidneys, and central nervous system.^1–4^ Recent studies have shown that multiple epigenetic regulatory mechanisms contribute to the pathogenesis of SLE.^5–8^

MicroRNAs (miRNAs) regulate target gene expression by binding to the 3′-untranslated region (3′-UTR) of target messenger RNAs (mRNAs), leading to either translational repression or degradation.^9^ Recent studies have shown that miRNAs regulate the function of both the innate and adaptive immune systems, are involved in various immune pathways, and serve as potential disease biomarkers and therapeutic targets.^10–12^ Altered expression of miRNAs is closely related to the occurrence of autoimmune diseases, including SLE,^13–16^ rheumatoid arthritis,^17–19^ and multiple sclerosis.^20–22^ We previously reported that miR-142-3p specifically targets interleukin-10 (IL-10) and CD84, which are members of the signaling lymphocytic activation molecule (SLAM) family, and that miR-142-5p specifically targets SLAM-associated protein (SAP) by interacting with its 3′-UTR. Inhibition of miR-142-3p/5p in healthy CD4^+^ T cells led to the increased expression of IL-10, CD84, and SAP as well as T cell overactivation and B cell hyperstimulation. According to a study, the expression of miR-142-3p/5p was significantly downregulated in SLE CD4^+^ T cells compared with that in healthy controls, and the overexpression of miR-142-3p/5p in SLE CD4^+^ T cells decreased IL-10, CD84, and SAP expression; reduced T cell activity; and decreased IgG production.^14^ However, the molecular mechanism by which miR-142-3p/5p expression in SLE CD4^+^ T cells is reduced remains to be elucidated.

The B cell lymphoma 6 protein (BCL-6) is an evolutionarily conserved zinc finger transcription factor that contains an N-terminal POZ/BTB domain. BCL-6 is expressed in almost all cell types but most highly expressed in B and T cells of the germinal center reaction. In T cells, BCL-6 is critical for T follicular helper (TFH) cell formation and acts as an obligatory regulator of commitment through repressing other T cell lineages, including Th1, Th2, Th9, and Th17 cells.^23–26^ BCL-6 can interact with different corepressors and histone deacetylases to repress the transcriptional expression of target genes.^27–29^ Basso et al.^30^ used ChIP-on-chip to reveal that BCL-6 can bind to the miR-142 promoter region in B cells. Thus, we suspected that the decreased miR-142-3p/5p expression in SLE CD4^+^ T cells was related to the abnormal expression of BCL-6.

Therefore, in this study, we demonstrate that the expression of BCL-6 is upregulated in SLE CD4^+^ T cells compared with that in normal controls. Excessive BCL-6 can recruit enhancer of zeste homolog 2 (EZH2) and histone deacetylase 5 (HDAC5) to the miR-142 promoter, increase H3K27 methylation and decrease H3K9/K14 acetylation, and then inhibit miR-142-3p/5p expression. Taken together, these results provide an explanation for the decreased miR-142-3p/5p expression in SLE CD4^+^ T cells observed in this study.

## Materials and methods

### Subjects

Thirty female SLE patients were recruited from outpatient clinics in the Second and Third Xiangya Hospital of Central South University. All patients fulfilled at least four of the SLE classification criteria set forth by the American College of Rheumatology.^31^ Relevant clinical and laboratory information regarding the patients is shown in Table 1. Lupus disease activity was assessed using the SLE Disease Activity Index (SLEDAI)^32^ Thirty female healthy controls were recruited from the medical staff at the Second and Third Xiangya Hospital. This study was approved by the Human Ethics Committee of the Second and Third Xiangya Hospital of Central South University, and written informed consent was obtained from all subjects.

**Table 1:** Clinical and laboratory characteristics of the patients with SLE used in the study

Characteristic	Characteristics SLE (*n* = 30)
Sex [male/female (*n*)]	0/30
Age (years) [median (range)]	33 (17–59)
SLEDAI score [median (range)]	12 (8–24)
Anti-dsDNA (IU/ml) [median (range)]	391.9 (57.9–825)
C3 (g/l) [median (range)]	0.87 (0.38–1.86)
C4 (g/l) [median (range)]	0.09 (0.07–0.18)
*Medication use (%)*	
Prednisolone or methylprednisolone	30
Hydroxychloroquine	25
Cyclosporine A	5
Mycophenolate mofetil	4

*SLE* systemic lupus erythematosus, *SLEDAI* SLE Disease Activity Index

### Isolation, culture, activation, and transfection of CD4^+^ T cells

CD4^+^ T cells were purified from 60 ml of venous peripheral blood using human CD4 beads according to the protocols provided by the manufacturer (Miltenyi, Bergisch Gladbach, Germany) and cultured in human T cell culture medium (Lonza, Walkersville, MD, USA). Purified normal CD4^+^ T cells were cultured in 24-well plates (1 × 10^6^/ml) and stimulated with plate-bound anti-CD3 antibody (eBioscience, CA, USA), followed by the addition of soluble anti-CD28 antibody (eBioscience) and incubation at 37 °C for 24 h. CD4^+^ T cells were transfected with the pCMV6 gene expression plasmid or the pRS gene interference plasmid using a Human T cell Nucleofector Kit and Amaxa nucleofector (Lonza). Briefly, CD4^+^ T cells were harvested and resuspended in 100 µl of human T cell nucleofector solution and then mixed with plasmid. The mixture was then used to electrotransfect T cells using the nucleofector program V-024 in the Amaxa nucleofector. The transfected cells were cultured in human T cell culture medium and harvested after 48 h.

### Flow cytometric analysis

CD4^+^ T cell suspensions (2 × 10^5^ cells) were incubated with FITC-conjugated anti-human CD25 antibody and APC-conjugated anti-human CD69 antibody (Becton Dickinson, NJ, USA) for 30 min at room temperature, washed twice with 2 ml of PBS containing 1% BSA and centrifuged at 400 × *g* for 5 min. The collected cells were resuspended in 0.5 ml of PBS/BSA. Data were acquired with a FACSCanto II flow cytometer (Becton Dickinson) and analyzed using FlowJo software (Becton Dickinson).

### RNA isolation and real-time PCR

Total RNA was isolated from CD4^+^ T cells using TRIzol reagent (Thermo Fisher Scientific, MA, USA). Complementary DNAs (cDNAs) were synthesized from 1 μg of total RNA using a miScript II RT Kit (Qiagen, CA, USA). DNA was synthesized from cDNA using a miScript SYBR Green PCR Kit (Qiagen). Real-time PCR was performed in triplicate using an ABI Prism 7500 instrument (Thermo Fisher Scientific). The expression of target miRNA was normalized to RNU6-2 miRNA expression, and the expression of mRNA was normalized to GAPDH mRNA expression. Fold-changes were calculated using the 2^−ΔΔCt^ method with the following formula: ΔΔCt = (Ct_target gene_−Ct_internal control_)_sample_−(Ct_target gene_−Ct_internal control_)_control_. Primers used to amplify miR-142-3p/5p and RNU6-2 were purchased from Qiagen. The sequences of the primers used to amplify mRNAs are listed in Table 2.

**Table 2:** Primer sequences used for real-time PCR

	Forward primer	Reverse primer
BCL-6	GCCGGCTGACAGCTGTATC	CGGAGACGATTAAGGTTGAGAA
CD40L	CACCCCCTGTTAACTGCCTA	CTGGATGTCTGCATCAGTGG
ICOS	ACAAAAGGAAGTGGAAACACAG	GCATGAGAATGGTCCAAGTTGT
IL-21	TGGTCCCTGAATTTCTGCC	TGTTTCTGTCTTCTCCCTGCA
GAPDH	AAGAGCTACGAGCTGCCTGAC	ATGGCCCAGCGGATGAG

### Western blot analysis

CD4^+^ T cells were lysed in protein lysis buffer containing proteinase inhibitor (Thermo Fisher Scientific). The lysates were centrifuged for 15 min at 14,000 × *g* at 4 °C, and the protein concentration was determined by Bradford protein assay (Thermo Fisher Scientific). Proteins were separated by SDS-PAGE using 10% polyacrylamide gels and then transferred onto PVDF membranes (Millipore, MA, USA). The membranes were blocked with 5% nonfat dry milk in Tris-buffered saline containing 0.1% Tween-20 (TBST) buffer and immunoblotted with anti-BCL-6 (Cell Signaling, BSN, USA), anti-CD40L (Abcam, Cambridge, UK), anti-ICOS (Abcam), and anti-GAPDH (Cell Signaling) primary antibodies. Band intensities were quantified using Quantity One software (Bio-Rad, CA, USA).

### Chromatin immunoprecipitation

Chromatin immunoprecipitation (ChIP) analysis was performed according to the instructions provided by a ChIP assay kit (Millipore). In brief, CD4^+^ T cells were fixed for 10 min at room temperature with 1% formaldehyde. The formaldehyde was quenched by the addition of glycine to a final concentration of 0.125 M. The cells were pelleted by centrifugation at 1500 rpm for 5 min, and the pellets were washed twice with 20 ml ice-cold PBS and then lysed. The pellet and resuspended lysates were sonicated to break the DNA into 500–1000 base pair fragments. Two micrograms of anti-BCL-6, anti-H3K27me3, anti-H3K9ac, anti-H3K14ac, anti-EZH2, anti-HDAC5, or control rabbit IgG antibody were added and incubated overnight at 4 °C while rotating. All antibodies were obtained from Cell Signaling. The immune complexes were precipitated with protein A agarose beads, washed, and then eluted in 100 μl of TE buffer containing 0.5% SDS and 200 μg/ml proteinase K. Precipitated DNA was further purified with spin columns before amplification of the target DNA by real-time PCR. The following primer pairs were used for real-time PCR: Forward 1 (−186 to −164): 5′-GAGGGAGGTAGAGGAGGCAAGT-3′ and Reverse 1 (+59 to +87): 5′-CACAGTACACTCATCCATAAAGTAGGAA-3′, Forward 2 (−375 to −350): 5′-GGGAGCTGTGGCTGCCTCATTTGGA-3′ and Reverse 2 (−193 to −170): 5′-CTCCTCTACCTCCCTCCCCCAC-3′, Forward 3 (−1200 to −1178): 5′-TTCGGAGCCTGGAGCACAGGGC-3′ and Reverse 3 (−943 to −921): 5′-CAGCCCTCCTCCTTGGACACCT-3′, and Forward 4 (−1840 to −1816): 5′-TGAACACCTAGCCTAGTGCCATCA-3′ and Reverse 4 (−1648 to −1627): 5′-AACTCCCTTCCCTTCCCAACA-3′.

### Coimmunoprecipitation

Nuclear proteins were extracted from CD4^+^ T cells using NE-PER™ Nuclear and Cytoplasmic Extraction Reagents (Thermo Fisher Scientific). Nuclear extracts were incubated overnight with anti-BCL-6 or control rabbit IgG antibody at 4 °C. Then, 30 μl of ChIP-grade Protein A/G Plus agarose beads (Millipore) was added to each IP reaction and incubated for 2 h at 4 °C while rotating. The protein A/G Plus-agarose beads were pelleted by centrifugation at 6000 rpm for 1 min, and the supernatant was removed. The precipitated complex was washed three times. The proteins were then eluted from the solid support using SDS sample loading buffer. The complexes were analyzed by western blotting with the primary antibodies anti-BCL-6, anti-EZH2, and anti-HDAC5.

### T and B cell costimulation assays

B cells were isolated by positive selection using CD19 magnetic beads (Miltenyi). At 48 h post-transfection, CD4^+^ T cells were co-cultured with autologous B cells at a ratio of 1:4 in 96-well round-bottomed plates according to previously described procedures.^33^ The medium was supplemented on day 4, and the supernatants were collected on day 8 to measure the IgG concentrations.

### Enzyme-linked immunosorbent assay

The IL-21 concentration was measured using a human IL-21 ELISA kit (Abcam, Cambridge, UK), and the IgG concentration was measured using a human IgG ELISA kit (Abcam) according to the manufacturer’s instructions. Three replicate wells were quantified for every sample, and all experiments were performed in triplicate. Optical density (OD) values at 450 nm were read using an ELx800 Absorbance Microplate Reader (BioTek, VT, USA).

### Statistical analysis

The results are expressed as the mean ± SD. Variables were compared using Student’s *t*-test (data from different transfections were compared by paired *t*-test, and other data were compared by two-group *t*-test). Correlations were analyzed using Pearson’s correlation coefficient. Significance was indicated by *P* ≤ 0.05. All analyses were performed using SPSS 19.0 software.

## Results

### Expression levels of BCL-6 in CD4^+^ T cells from SLE patients and healthy controls

**Fig. 1:**
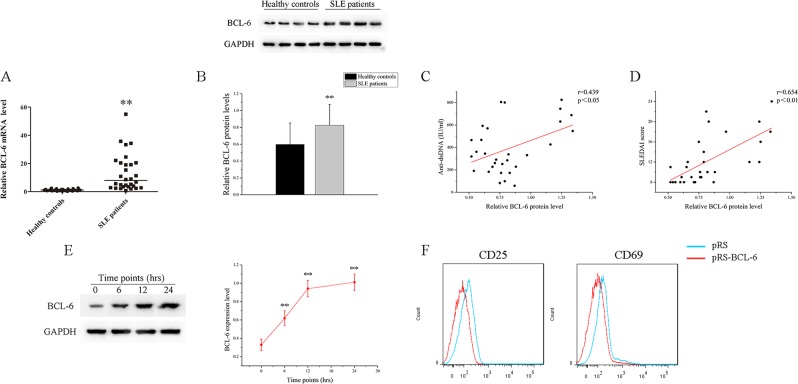
Expression level of BCL-6 in CD4^+^ T cells from systemic lupus erythematosus (SLE) patients and healthy controls. **a** Relative *BCL-6* mRNA levels in CD4^+^ T cells isolated from healthy controls or SLE patients (*n* = 30 for each group) were assessed by real-time PCR and normalized to those of GAPDH. **b** BCL-6 protein expression in CD4^+^ T cells isolated from healthy controls or SLE patients (*n* = 30 for each group) was analyzed by western blot analysis. One representative blot is shown (upper panel). The intensities of the bands were semiquantitated and normalized to that of the band for GAPDH (lower panel). **c** The correlation between BCL-6 protein level and anti-dsDNA titer (*n* = 30). **d** The correlation between BCL-6 protein level and SLE Disease Activity Index (SLEDAI) score (*n* = 30). **e** Representative western blot showing BCL-6 expression levels in normal CD4^+^ T cells after activation of the TCR (left panel). The intensities of the bands were semiquantitated and normalized to that of the band for GAPDH (right panel). Data represent the mean of three independent experiments. **f** Histograms showing CD4^+^ T cells expressing CD25 and CD69 as determined by flow cytometry (**P < 0.01)

Our previous studies have shown that miR-142-3p/5p expression levels are downregulated in CD4^+^ T cells from SLE patients compared with those in CD4^+^ T cells from healthy individuals.^14^ Here, we investigated whether BCL-6 is involved in the molecular mechanism by which the expression of miR-142-3p/5p is reduced in the CD4^+^ T cells of SLE patients. First, we detected the expression levels of BCL-6 in CD4^+^ T cells from SLE patients and healthy controls. Real-time PCR and western blotting showed that BCL-6 expression was significantly upregulated in SLE CD4^+^ T cells compared with that in healthy controls (Fig. 1a, b). We analyzed the correlation between BCL-6 expression and clinicopathological parameters. The expression level of BCL-6 was positively correlated with the anti-dsDNA titer and SLEDAI score (Fig. 1c, d).

Furthermore, we detected the expression level of BCL-6 after activation of the TCR. We observed a significant increase in BCL-6 expression in normal CD4^+^ T cells after their stimulation with anti-CD3/CD28 antibody (Fig. 1e). To determine the relationship between BCL-6 and the activation of CD4^+^ T cells, normal CD4^+^ T cells were transfected with the *BCL-6* interference plasmid pRS-*BCL-6* or the negative control plasmid pRS. After 24 h of transfection, CD4^+^ T cells were activated by anti-CD3/CD28 antibodies for 12 h. The expression of CD25 and CD69 was detected by flow cytometry. The downregulation of BCL-6 expression in normal CD4^+^ T cells significantly reduced the expression of CD25 and CD69 induced by TCR stimuli (Fig. 1f). In summary, BCL-6 may be involved in the pathogenesis of SLE as a promoter of CD4^+^ T cell activation.

### Upregulated BCL-6 suppresses miR-142-3p/5p expression in SLE CD4^+^ T cells

**Fig. 2:**
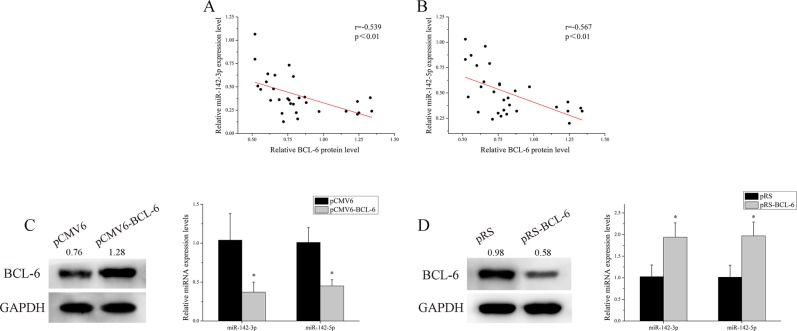
Upregulated BCL-6 suppresses miR-142-3p/5p expression in SLE CD4^+^ T cells. **a** The correlation between miR-142-3p expression and BCL-6 protein level in SLE CD4^+^ T cells (*n* = 30). **b** The correlation between miR-142-5p expression and BCL-6 protein levels in SLE CD4^+^ T cells (*n* = 30). **c** Representative western blot showing BCL-6 expression levels in normal CD4^+^ T cells transfected with BCL-6 expression plasmid or control plasmid (left panel). Relative miR-142-3p/5p levels in normal CD4^+^ T cells transfected with BCL-6 expression or control plasmid were assessed by real-time PCR and normalized to the levels of RNU6-2 (right panel). Data represent the mean of three independent experiments. **d** Representative western blot showing BCL-6 expression levels in SLE CD4^+^ T cells transfected with *BCL-6* interference plasmid or control plasmid (left panel). Relative miR-142-3p/5p levels in SLE CD4^+^ T cells transfected with *BCL-6* interference plasmid or control plasmid were assessed by real-time PCR and normalized to the levels of RNU6-2 (right panel). Data represent the mean of three independent experiments (*P < 0.05)

To explore the relationship between BCL-6 and miR-142-3p/5p expression, we measured the expression levels of miR-142-3p/5p in SLE CD4^+^ T cells by real-time PCR and performed correlation analysis between BCL-6 and miR-142-3p/5p expression in SLE CD4^+^ T cells. BCL-6 and miR-142-3p/5p expression levels showed a significantly negative correlation (Fig. 2a, b).

To investigate the effect of BCL-6 on miR-142-3p/5p expression in CD4^+^ T cells, we transfected normal CD4^+^ T cells with the *BCL-6* expression plasmid pCMV6-*BCL-6* and SLE CD4^+^ T cells with the *BCL-6* interference plasmid pRS-*BCL-6*. The expression of miR-142-3p/5p was downregulated significantly in normal CD4^+^ T cells overexpressing BCL-6 compared with that in the negative control (Fig. 2c), and miR-142-3p/5p expression was upregulated significantly in SLE CD4^+^ T cells in which BCL-6 expression had been knocked down compared with that in the negative control (Fig. 2d). Taken together, our results suggested that the increased expression of BCL-6 in CD4^+^ T cells is one of the main causes of the reduced expression of miR-142-3p/5p.

### Upregulated BCL-6 binds more strongly to the miR-142 promoter in SLE CD4^+^ T cells

**Fig. 3:**
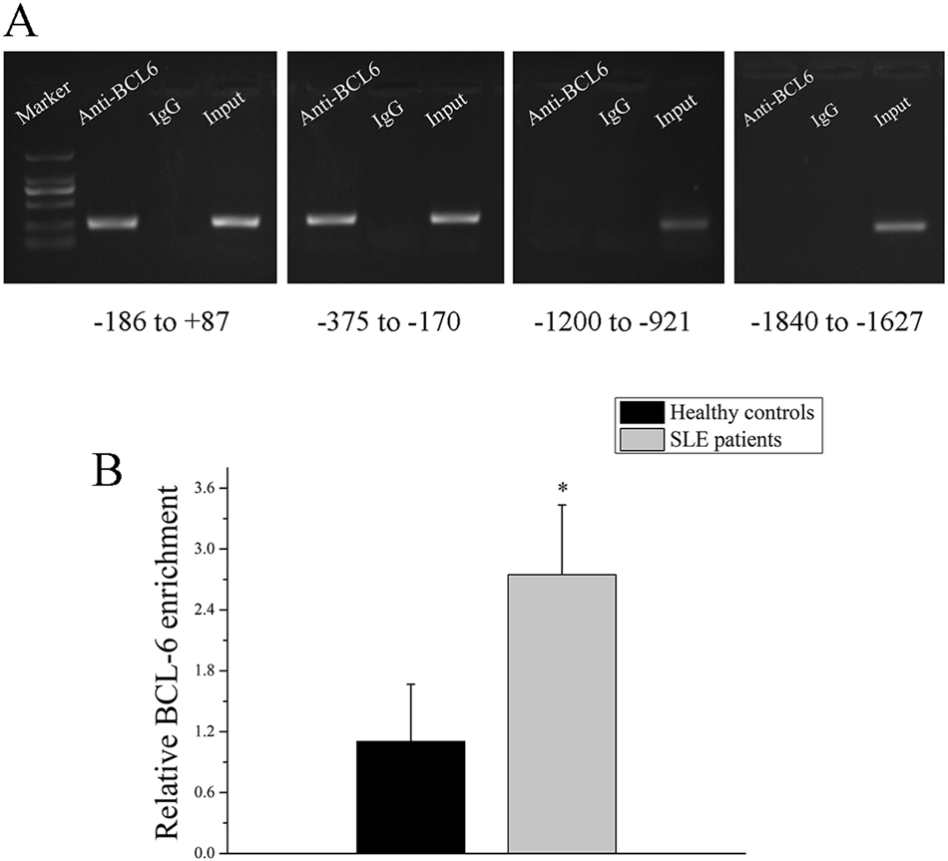
Upregulated BCL-6 binds more strongly to the miR-142 promoter in SLE CD4^+^ T cells than in CD4^+^ T cells from healthy controls. **a** ChIP-PCR showed that BCL-6 binds to the promoter proximal element (−375 to +87) of miR-142 in CD4^+^ T cells. Data represent the mean of three independent experiments. **b** ChIP-qPCR analysis of the enrichment of BCL-6 bound to the miR-142 promoter in chromatin fractions extracted from CD4^+^ T cells from healthy controls (*n* = 12) or SLE patients (*n* = 12). The results are presented relative to those obtained with input DNA prepared from untreated chromatin (*P < 0.05)

To confirm whether BCL-6 can directly bind to the miR-142 promoter, ChIP-PCR analysis of SLE CD4^+^ T cells using an anti-BCL-6 antibody was performed. Several pairs of primers that covered the miR-142 promoter +87 to −1840 region were used. The results showed that BCL-6 binds to the promoter proximal element (−375 to +87) of miR-142 in CD4^+^ T cells (Fig. 3a).

To confirm that the overexpressed BCL-6 binds more strongly to the miR-142 promoter in the CD4^+^ T cells of SLE patients than to that in the CD4^+^ T cells of healthy controls, ChIP-qPCR analysis using anti-BCL-6 antibody was performed. As shown in Fig. 3b, the binding of BCL-6 to the miR-142 promoter was stronger in the CD4^+^ T cells of SLE patients than in those of healthy controls.

### BCL-6 regulates miR-142-3p/5p expression through altering the histone H3K27me3 and H3K9/K14ac of its promoter

**Fig. 4:**
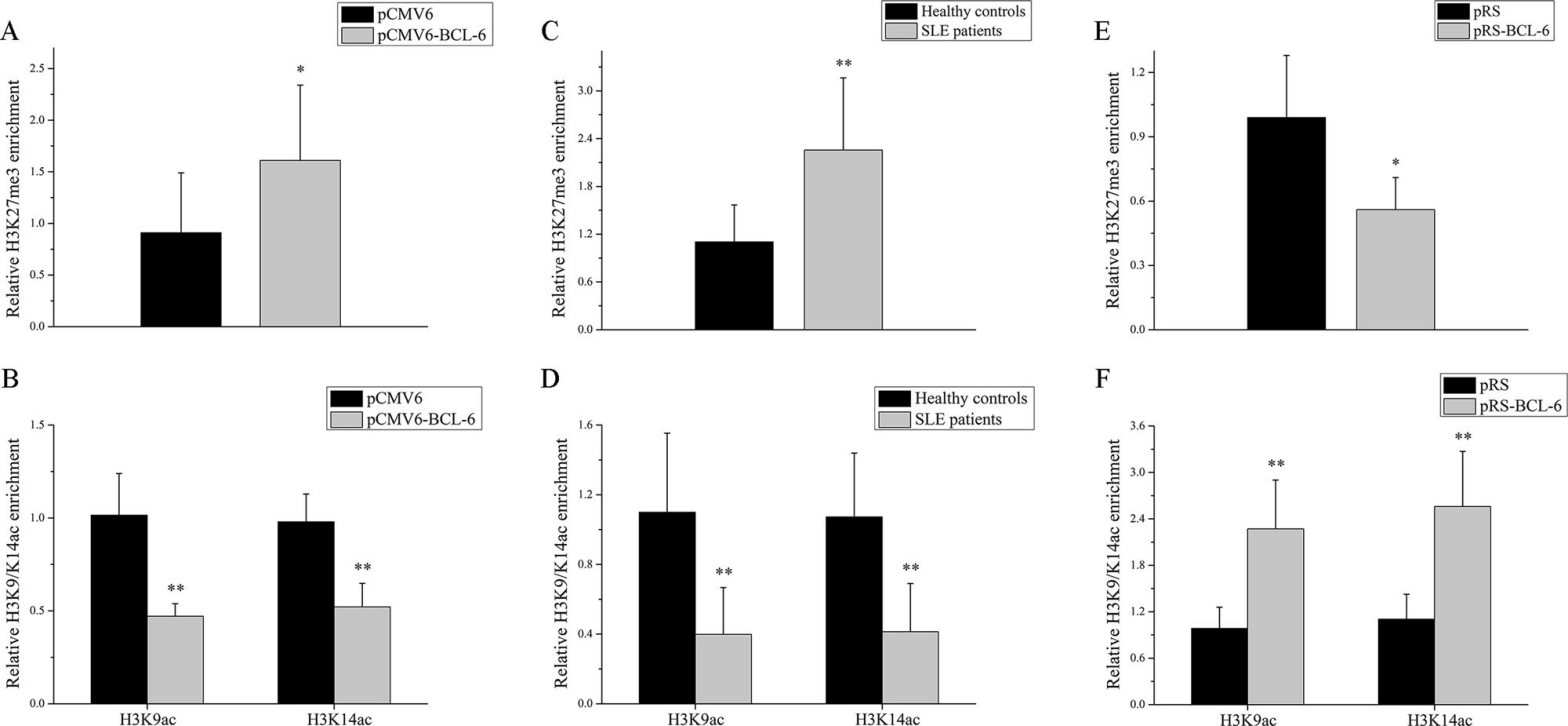
BCL-6 regulates histone H3K27me3 and H3K9/K14ac levels in the miR-142 promoter. **a**, **b** ChIP-qPCR analysis of the enrichment of histone H3K27me3 (**a**) and H3K9/K14ac (**b**) in the miR-142 promoter in chromatin fractions extracted from normal CD4^+^ T cells transfected with *BCL-6* expression plasmid or negative control plasmid. The results are presented relative to those obtained with input DNA prepared from untreated chromatin. Data represent the mean of three independent experiments. **c**, **d** ChIP-qPCR analysis of the enrichment of histone H3K27me3 (**c**) and H3K9/K14ac (**d**) in the miR-142 promoter in chromatin fractions extracted from normal (*n* = 12) and SLE (*n* = 12) CD4^+^ T cells. The results are presented relative to those obtained with input DNA prepared from untreated chromatin. **e**, **f** ChIP-qPCR analysis of the enrichment of histone H3K27me3 (**e**) and H3K9/K14ac (**f**) in the miR-142 promoter in chromatin fractions extracted from SLE CD4^+^ T cells transfected with *BCL-6* interference plasmid or negative control plasmid. The results are presented relative to those obtained with input DNA prepared from untreated chromatin. Data represent the mean of three independent experiments (*P < 0.05, **P < 0.01)

Histone modification is a critical factor in regulating gene expression. To further demonstrate the molecular mechanism by which BCL-6 inhibits miR-142-3p/5p expression, we detected histone modifications of the miR-142 promoter in normal CD4^+^ T cells transfected with *BCL-6* expression plasmid or negative control by ChIP-qPCR. Interestingly, the ectopic expression of BCL-6 in normal CD4^+^ T cells increased histone H3 trimethylation at Lys 27 (H3K27me3) and decreased histone H3 acetylation at Lys 9 (H3K9ac) and Lys14 (H3K14ac) of the miR-142 promoter (Fig. 4a, b).

Furthermore, we detected H3K27me3 and H3K9/K14ac of the miR-142 promoter in CD4^+^ T cells from 12 SLE patients and 12 healthy controls. ChIP-qPCR showed that the levels of H3K27me3 of the miR-142 promoter in SLE patients were significantly higher than those in healthy controls (Fig. 4c), and the levels of H3K9/K14ac of the miR-142 promoter in SLE patients were significantly lower than those in healthy controls (Fig. 4d). In addition, SLE CD4^+^ T cells were transfected with the *BCL-6* interference plasmid or negative control plasmid. The H3K27me3 level was decreased (Fig. 4e), and the H3K9/K14ac level was increased in the promoter region of miR-142 in SLE CD4^+^ T cells after BCL-6 interference (Fig. 4f). Together, our results suggest that BCL-6 suppresses the expression of miR-142-3p/5p by increasing H3K27me3 levels and reducing H3K9/K14ac levels in CD4^+^ T cells.

### BCL-6 recruits EZH2 and HDAC5, which bind to the miR-142 promoter

**Fig. 5:**
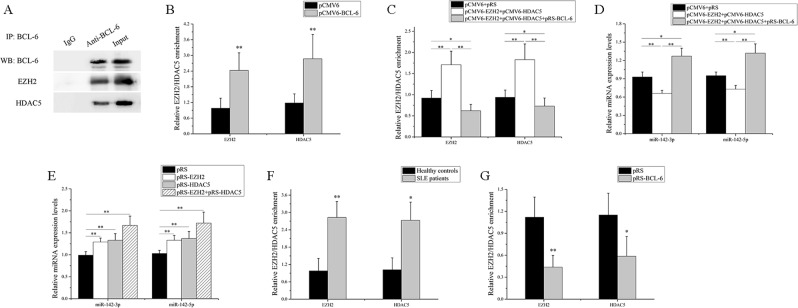
BCL-6 recruits EZH2 and HDAC5, which bind to the miR-142 promoter. **a** Coimmunoprecipitation using anti-BCL-6 in SLE CD4^+^ T cells and the detection of BCL-6 and EZH2/HDAC5 by western blotting. **b** ChIP-qPCR analysis of the enrichment of EZH2 and HDAC5 bound to the miR-142 promoter in chromatin fractions extracted from normal CD4^+^ T cells transfected with *BCL-6* expression plasmid or a negative control. The results are presented relative to those obtained with input DNA prepared from untreated chromatin. Data represent the mean of three independent experiments. **c** ChIP-qPCR analysis of the enrichment of EZH2 and HDAC5 bound to the miR-142 promoter in chromatin fractions extracted from normal CD4^+^ T cells transfected with *EZH2/HDAC5* expression plasmid and *BCL-6* interference plasmid. Data represent the mean of three independent experiments. **d** Real-time PCR analysis of the expression levels of miR-142-3p/5p in normal CD4^+^ T cells transfected with *EZH2/HDAC5* expression plasmid and BCL-6 interference plasmid. Data represent the mean of three independent experiments. **e** Real-time PCR analysis of the expression levels of miR-142-3p/5p in normal CD4^+^ T cells transfected with *EZH2/HDAC5* interference plasmid. Data represent the mean of three independent experiments. **f** ChIP-qPCR analysis of the enrichment of EZH2 and HDAC5 bound to the miR-142 promoter in chromatin fractions extracted from normal (*n* = 12) and SLE (*n* = 12) CD4^+^ T cells. **g** ChIP-qPCR analysis of the enrichment of EZH2 and HDAC5 bound to the miR-142 promoter in chromatin fractions extracted from SLE CD4^+^ T cells transfected with BCL-6 interference plasmid or negative control. Data represent the mean of three independent experiments (*P < 0.05, **P < 0.01)

To explain how BCL-6 regulates histone methylation and acetylation, we focused on EZH2, a histone H3K27 methyltransferase. Furthermore, we used the STRING database (https://string-db.org/cgi/input.pl) to identify proteins that might interact with BCL-6, which included TP53, FOXO3, and HDAC5. HDAC5 is closely related to histone deacetylation. We tested whether BCL-6 can form complexes with EZH2 and HDAC5. As shown in Fig. 5a, coimmunoprecipitation experiments in SLE CD4^+^ T cells showed that BCL-6 coprecipitates with EZH2 as well as HDAC5. Furthermore, normal CD4^+^ T cells were transfected with the BCL-6 expression plasmid pCMV6-*BCL-6* or negative control plasmid. EZH2 and HDAC5 binding to the promoter region of miR-142 was increased after BCL-6 expression (Fig. 5b). The binding of EZH2 and HDAC5 to the miR-142 promoter region was increased after the overexpression of EZH2 and HDAC5 in normal CD4^+^ T cells compared with that in the negative control group (Fig. 5c). However, when BCL-6 expression was knockdown, the binding of EZH2 and HDAC5 to the miR-142 promoter region was significantly lower than that in the negative control group (Fig. 5c). The expression levels of miR-142-3p/5p were decreased significantly after the overexpression of EZH2 and HDAC5 in normal CD4^+^ T cells (Fig. 5d). Interestingly, the expression of miR-142-3p/5p increased slightly after the overexpression of EZH2 and HDAC5, which simultaneously inhibited the expression of BCL-6, compared with that in the negative control group. (Fig. 5d). In addition, the inhibition of EZH2 and/or HDAC5 expression in SLE CD4^+^ T cells significantly increased the expression levels of miR-142-3p/5p (Fig. 5e).

We also detected the binding of EZH2 and HDAC5 to the promoter region of miR-142 in CD4^+^ T cells from 12 SLE patients and 12 healthy controls by ChIP-qPCR. As shown in Fig. 5f, the binding of EZH2 and HDAC5 to the miR-142 promoter was stronger in SLE patients than in healthy controls. Furthermore, SLE CD4^+^ T cells were transfected with the BCL-6 interference plasmid or the negative control plasmid. EZH2 and HDAC5 binding to the miR-142 promoter was decreased after BCL-6 knockdown (Fig. 5g). These data strongly suggest that BCL-6 plays a crucial role in recruiting EZH2 and HDAC5 to the miR-142 promoter.

### Knockdown of BCL-6 expression alleviates the self-reactivity of SLE CD4^+^ T cells

**Fig. 6:**
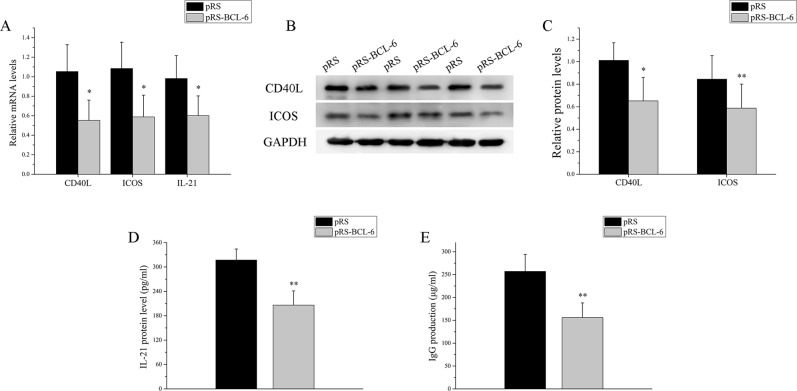
BCL-6 knockdown alleviates the self-reactivity of SLE CD4^+^ T cells. **a** Relative *CD40L*, *ICOS*, and *IL-21* mRNA levels in SLE CD4^+^ T cells transfected with *BCL-6* interference plasmid or negative control were assessed by real-time PCR and normalized to the mRNA levels of GAPDH. **b**, **c** CD40L and ICOS expression levels in SLE CD4^+^ T cells transfected with *BCL-6* interference plasmid or negative control were analyzed by western blotting. One representative blot is shown (**b**). The intensities of the bands were semiquantitated and normalized to that of the band for GAPDH **c**. **d** SLE CD4^+^ T cells transfected with *BCL-6* interference plasmid or negative control. The concentrations of IL-21 in the cell culture supernatant after 48 h post-transfection were measured by ELISA. **e** SLE CD4^+^ T cells were transfected with *BCL-6* interference plasmid or negative control, and the transfected cells were co-cultured with purified autologous B cells, following which IgG levels in the culture supernatants were measured by ELISA. Data represent the mean of three independent experiments per group (*P < 0.05, **P < 0.01)

To determine the effect of the downregulation of BCL-6 expression on SLE CD4^+^ T cell function, BCL-6 expression was knocked down in SLE CD4^+^ T cells, and the mRNA and protein expression levels of CD40L, ICOS, and IL-21 in SLE CD4^+^ T cells were then detected. As expected, we observed a significant decrease in CD40L, ICOS, and IL-21 expression levels (Fig. 6a, d). Furthermore, SLE CD4^+^ T cells in which BCL-6 expression was knocked down were co-cultured with purified autologous B cells. IgG levels in the culture supernatants were measured by enzyme-linked immunosorbent assay (ELISA). As shown in Fig. 6e, IgG antibody production in the BCL-6 interference group was obviously lower than that in the negative control group. Together, these results suggest that the knockdown of BCL-6 expression inhibits the self-reactivity of SLE CD4^+^ T cells, which can reduce the autoantibody production of B cells.

## Discussion

SLE is characterized by autoantigen-driven, T cell-dependent autoimmune disease leading to autoantibody production. Abnormalities in the function, regulation, and interactions of immune cells, such as those caused by the overexpression of the costimulators CD70, CD40L, CD84, and SAP in SLE CD4^+^ T cells, induce autoantibody production in B cells, the loss of self-tolerance, and the synthesis of proinflammatory cytokines, such as IL-4, IL-6, and IL-10. These cytokines in turn lead to immune-mediated inflammatory pathogenesis and multiple organ injuries.^3,14,34–36^ In our previous study, we demonstrated that miR-142-3p/5p directly inhibits SAP, CD84, and IL-10 translation. Furthermore, the expression of miR-142-3p/5p was significantly downregulated in SLE CD4^+^ T cells compared with that in healthy controls. In addition, the expression levels of miRNA-142-3p/5p in healthy CD4^+^ T cells after stimulation with the anti-CD3/CD28 antibody showed a decreasing trend, but this decrease was not obvious or statistically significant.^14^ In this study, we confirmed that the increased expression of BCL-6 strongly increases binding to the miR-142 promoter proximal element and the inhibition of miR-142-3p/5p expression by increasing H3K27me3 levels and reducing H3K9/K14ac levels in SLE CD4^+^ T cells. We also found that anti-CD3/CD28 antibody stimulation could significantly upregulate the expression of BCL-6 in normal CD4^+^ T cells. Combined with the results of previous studies, our results were puzzling, and we wondered how CD3/CD28 activation can significantly upregulate the expression of BCL-6 in normal CD4^+^ T cells without obviously downregulating miRNA-142-3p/5p. Therefore, we examined the enrichment of BCL-6, EZH2, HDAC5, H3K27me3, and H3K9/14ac in the miRNA-142 promoter in normal CD4^+^ T cells before and after the activation of CD3/CD28. The levels of BCL-6, EZH2, and H3K27me3 were increased, while the levels of HDAC5 and H3K9/14ac in the miRNA-142 promoter were not significantly different in normal CD4^+^ T cells before and after CD3/CD28 stimulation (data not shown). These results provide some explanations but do not fully reveal why an increase in BCL-6 caused by T cell activation did not significantly reduce the expression of miRNA-142-3p/5p. We speculate that the downstream effects caused by CD3/CD28 activation are complex and that some factors that upregulate the expression of miRNA-142-3p/5p may be activated, partially offsetting the downregulation of miRNA-142-3p/5p induced by BCL-6/EZH2, resulting in the slight downregulation of miRNA-142-3p/5p expression. Generally, these data suggest that the increased expression of BCL-6 is one of the main causes of the reduced expression of miR-142-3p/5p in CD4^+^ T cells from SLE patients.

When H3K27 is trimethylated, it is tightly associated with inactive gene promoters. Because of its dramatic and predictable effects on gene expression, H3K27me3 has become a favorite tool for epigenetic researchers looking for inactive genes. Most histone methylations are catalyzed by several enzymes. H3K27me3 is distinct in that it involves only one known methyltransferase: EZH2.^37^ EZH2, a catalytic subunit of polycomb repressive complex 2 (PRC2), trimethylates Lys 27 on histone H3, repressing gene transcription.^38,39^ Beguelin et al.^23^ found that BCL-6 and EZH2 cooperate to recruit a noncanonical PRC1-BCOR-CBX8 complex that binds to H3K27me3 at bivalent promoters and then represses the expression of differentiation-related genes in germinal center B cells and promotes lymphomagenesis. Recent studies have found that EZH2 plays an important role in T cell plasticity, activation, and differentiation.^40–42^ EZH2 may participate in the pathogenesis of SLE by epigenetic remodeling and upregulate or downregulate the expression of lupus-related genes.^42^ Overexpressed EZH2 in SLE CD4^+^ T cells mediates increased T cell adhesion to endothelial cells by downregulating JAM-A DNA methylation and upregulating its expression. The blockade of EZH2 or JAM-A might have therapeutic potential by reducing T cell adhesion, migration, and extravasation in patients with lupus.^43^ H3K9ac, an important histone acetylation modification, is highly correlated with active promoters. H3K9ac and H3K14ac showed a high co-occurrence, and H3K9ac and H3K14ac together are considered the hallmark of active gene promoters.^44^ HDAC5, which belongs to the class II histone deacetylase family, possesses histone deacetylase activity and represses transcription when tethered to a promoter. Gu et al. demonstrated that highly expressed HDAC5 was closely related to the downregulation of TAp63 and maspin in hepatocellular carcinoma (HCC) tissues and involved in HCC development. Knockdown of HDAC5 in HCC cells induced TAp63 expression, accompanied by increased H3K9ac levels at the TAp63 promoter.^45^ Our study demonstrated that BCL-6 can form complexes with EZH2 and HDAC5, mediating their binding to the miR-142 promoter. The increased binding of EZH2 and HDAC5 to the miR-142 promoter elevated H3K27me3 levels and reduced H3K9/K14ac levels in SLE CD4^+^ T cells. These findings elucidate the molecular mechanism by which BCL-6 mediates the decreased expression of miR-142-3p/5p in SLE CD4^+^ T cells.

TFH cells, a subset of T helper cells, specialize in helping B cells to become professional antibody producers.^46^ An increasing amount of evidence indicates the pathogenic role of TFH cells in human lupus; this evidence includes alterations in the phenotype of circulating TFH cells and increases in their population size.^47–49^ BCL-6, CD40L, ICOS, and IL-21 have been acknowledged as master regulators for the development and function of TFH cells. CD40L acts as a costimulatory molecule and is particularly important in stimulating TFH cells. CD40L promotes B cell maturation and function by engaging CD40 on the B cell surface, facilitating cell–cell communication.^50^ CD40L plays an important role in the pathogenesis of a number of autoimmune diseases.^51,52^ For example, CD40L expression was increased in CD4^+^ T cells in lupus, and CD40L then interacted with CD40 expressed on B cells, contributing to the overproduction of pathogenic autoantibodies.^53,54^ Inducible T cell costimulatory (ICOS), a costimulatory molecule in the CD28 superfamily, is expressed on all effector T cells, and signaling through ICOS–ICOSL remains critical for TFH generation and GC formation.^55,56^ In lupus, the number of circulating ICOS^+^ TFH cells is increased, and these cells are positively correlated with serum autoantibody titers and disease activity and/or severity.^25^ Production of high levels of IL-21 is a hallmark of TFH cells. IL-21 promotes TFH cell differentiation and therefore has an autocrine effect on T cells and participates in a positive feedback loop on naive CD4^+^ T cells. Above all, IL-21 promotes the differentiation of B cells into plasma cells and plays a critical role in regulating immunoglobulin production.^57,58^ Plasma levels of IL-21 are significantly elevated in SLE patients compared with those in healthy controls, and high concentrations of IL-21 are associated with disease severity and SLEDAI scores.^59^ In this study, we observed significantly decreased CD40L, ICOS, and IL-21 expression in SLE CD4^+^ T cells after BCL-6 expression was knocked down. The knockdown of BCL-6 expression in SLE CD4^+^ T cells clearly inhibited the function of TFH cells and weakened the interaction of T and B cells.

In summary, we found that the increased expression of BCL-6 plays an important role in inhibiting miR-142-3p/5p expression by regulating histone H3 methylation and acetylation of the miR-142 promoter in SLE CD4^+^ T cells. It is thus possible that inhibiting BCL-6 prevents T cell activation and the autoimmune response.
